# Supplementary material for: The Small RNA Universe of Capitella teleta
Source: Front Mol Biosci. 2022 Feb 25;9:802814. doi: 10.3389/fmolb.2022.802814 (PMC8915122; doi:10.3389/fmolb.2022.802814)
Supplement: Supplementary file 1 [file DataSheet1.ZIP › Supplement/known/CAPTEscaffold_189_13497.pdf]

Provisional ID	:	CAPTEscaffold_189_13497
Score total	:	1455.9
Score for star read(s)	:	3.9
Score for read counts	:	1445.5
Score for mfe	:	1.9
Score for randfold	:	1.6
Score for cons. seed	:	3
Total read count	:	2847
Mature read count	:	2714
Loop read count	:	0
Star read count	:	133

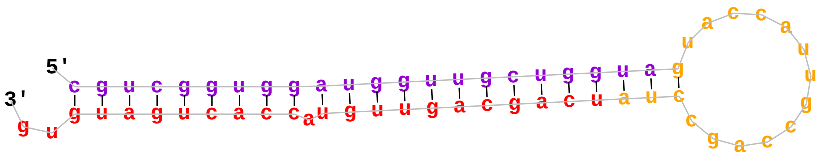

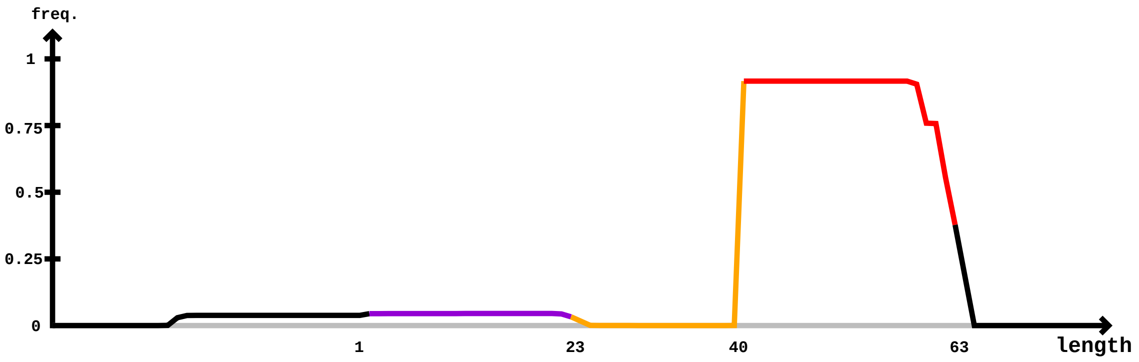

Star Mature

```
5'- gcgugcguauacaggaaugcuagcugugucucgucgguggaugguugcugguaguaccauugccagccuaucagcaguuguaccacugaugugacacgccuugcuucu  -3'  obs
    gcgugcguauacaggaaugcuagcugugucucgucgguggaugguugcugguaguaccauugccagccuaucagcaguuguaccacugaugugacacgccuugcuucu        exp
    gcgugcguauacaggaaugcuagcugugucucgucgguggaugguugcugguaguaccauugccagccuaucagcaguuguaccacugaugugacacgccuugcuucu        known
    .............((((.((.((.((((((.(((((((((((((((((((((((.............)))))))))))))).))))))))).)))))).)).))))))   reads   mm   sample
    ..........acaggaaugcuagcugugucu.............................................................................   2       0    seq
    ...........caggaaugcuagcugugucu.............................................................................   83      0    seq
    ...........caggaaugcuagcugugucC.............................................................................   1       1    seq
    ...........caCgaaugcuagcugugucu.............................................................................   1       1    seq
    ............aggaaugcuagcugugucA.............................................................................   1       1    seq
    ............aggaaugcuagcugugucu.............................................................................   24      0    seq
    .............ggaaugcuagcugugucu.............................................................................   1       0    seq
    ...............................cgucgguggaugguugcugg.........................................................   2       0    seq
    ...............................cgucgguggaugguugcugU.........................................................   3       1    seq
    ...............................cgucgguggaugguugcuggu........................................................   31      0    seq
    ...............................cgucgguggaugguugcAggu........................................................   1       1    seq
    ...............................cgucgguggaugguugcuggua.......................................................   91      0    seq
    ...............................cgAcgguggaugguugcuggua.......................................................   1       1    seq
    ...............................cgucgguAgaugguugcuggua.......................................................   1       1    seq
    ...............................cgucgAuggaugguugcuggua.......................................................   1       1    seq
    ...............................cgucgguggaugguugcugguaCu.....................................................   1       1    seq
    .................................ucgguggaugguugcuggua.......................................................   1       0    seq
    .........................................ugguugcugguaguacca.................................................   1       0    seq
    .....................................................................Gucagcaguuguaccacugaugug...............   1       1    seq
    ......................................................................ucagcaguuguaccacug....................   34      0    seq
    ......................................................................ucagcaguuguaGcacuga...................   1       1    seq
    ......................................................................ucagcaguuguaUcacuga...................   1       1    seq
    ......................................................................Acagcaguuguaccacuga...................   3       1    seq
    ......................................................................ucagcaguugAaccacuga...................   1       1    seq
    ......................................................................Gcagcaguuguaccacuga...................   1       1    seq
    ......................................................................uUagcaguuguaccacuga...................   1       1    seq
    ......................................................................ucagcaguuguaccacuga...................   422     0    seq
    ......................................................................ucagcaguuguaccacugG...................   3       1    seq
    ......................................................................ucagcaguuguaccacugau..................   4       0    seq
    ......................................................................ucagcaguAguaccacugaug.................   2       1    seq
    ......................................................................ucaCcaguuguaccacugaug.................   1       1    seq
    ......................................................................ucagcaguuguaccacugaug.................   589     0    seq
```

Star **Mature**

```
gcgugcguauacaggaaugcuagcugugucucgucgguggaugguugcugguaguaccauugccagccuaucagcaguuguaccacugaugugacacgccuugcuucu
......................................................................ucagcaguuguaccacuAaug.................   2      1      seq
......................................................................ucGgcaguuguaccacugaug.................   1      1      seq
......................................................................ucagcaguuguaccacugauA.................   1      1      seq
......................................................................ucagcaguuguaccacugaAg.................   1      1      seq
......................................................................ucagcaguuguaccacAgaug.................   2      1      seq
......................................................................ucagcaguuguaccacugaugu................   515    0      seq
......................................................................ucagcagAuguaccacugaugu................   1      1      seq
......................................................................ucagcaguuguGccacugaugu................   1      1      seq
......................................................................ucagcaguuguaUcacugaugu................   1      1      seq
......................................................................Acagcaguuguaccacugaugu................   7      1      seq
......................................................................ucaCcaguuguaccacugaugu................   1      1      seq
......................................................................Ncagcaguuguaccacugaugug...............   1      1      seq
......................................................................Gcagcaguuguaccacugaugug...............   2      1      seq
......................................................................ucagcaguuguaccacugauguU...............   2      1      seq
......................................................................ucagcaguuguaccacuAaugug...............   2      1      seq
......................................................................ucagcaguuguaccacugauguA...............   13     1      seq
......................................................................ucagcaguAguaccacugaugug...............   1      1      seq
......................................................................ucagcaguuguaccacugaugug...............   1065   0      seq
......................................................................Acagcaguuguaccacugaugug...............   4      1      seq
......................................................................uUagcaguuguaccacugaugug...............   1      1      seq
......................................................................ucagcaguuguaccacugGugug...............   1      1      seq
......................................................................ucagcaguuguaccacugauguC...............   1      1      seq
......................................................................ucagcaguugAaccacugaugug...............   1      1      seq
......................................................................ucagcaguuguaUcacugaugug...............   2      1      seq
......................................................................ucagcaguGguaccacugaugug...............   1      1      seq
......................................................................ucagcaguuguaccacugauguAa..............   1      1      seq
......................................................................ucagcaguuguaccacugauguga..............   11     0      seq
......................................................................ucagcaguuguaccacugaugugU..............   8      1      seq
```
